# Overexpression of *GhWRKY27a* reduces tolerance to drought stress and resistance to *Rhizoctonia solani* infection in transgenic *Nicotiana benthamiana*

**DOI:** 10.3389/fphys.2015.00265

**Published:** 2015-09-24

**Authors:** Yan Yan, Haihong Jia, Fang Wang, Chen Wang, Shuchang Liu, Xingqi Guo

**Affiliations:** State Key Laboratory of Crop Biology, College of Life Sciences, Shandong Agricultural UniversityTaian, China

**Keywords:** abscisic acid, cotton (*Gossypium hirsutum*), drought stress, *Rhizoctonia solani* infection, VIGS, WRKY transcription factor

## Abstract

WRKY proteins constitute transcriptional regulators involved in various biological processes, especially in coping with diverse biotic and abiotic stresses. However, in contrast to other well-characterized WRKY groups, the functions of group III WRKY transcription factors are poorly understood in the economically important crop cotton (*Gossypium hirsutum*). In this study, a group III WRKY gene from cotton, *GhWRKY27a*, was isolated and characterized. Our data indicated that GhWRKY27a localized to the nucleus and that *GhWRKY27a* expression could be strongly induced by abiotic stresses, pathogen infection, and multiple defense-related signaling molecules. Virus-induced gene silencing (VIGS) of *GhWRKY27a* enhanced tolerance to drought stress in cotton. In contrast, *GhWRKY27a* overexpression in *Nicotiana benthamiana* markedly reduced plant tolerance to drought stress, as determined through physiological analyses of leaf water loss, survival rates, and the stomatal aperture. This susceptibility was coupled with reduced stomatal closure in response to abscisic acid and decreased expression of stress-related genes. In addition, *GhWRKY27a*-overexpressing plants exhibited reduced resistance to *Rhizoctonia solani* infection, mainly demonstrated by the transgenic lines exhibiting more severe disease symptoms, accompanied by attenuated expression of defense-related genes in *N. benthamiana*. Taken together, these findings indicated that *GhWRKY27a* functions in negative responses to drought tolerance and in resistance to *R. solani* infection.

## Introduction

Due to their sessile growth habit, plants are constantly exposed to various biotic and abiotic stresses, such as pathogen infection and drought stress. To respond appropriately to these stresses, plants have evolved a highly sophisticated signaling network to perceive external signals and manifest adaptive responses with proper physiological and molecular changes (Asai et al., 2002; Smékalová et al., 2014). The transcriptional regulation of a multitude of defense-related genes is a key step during these processes. The regulation of these genes at the transcriptional level is largely mediated by the specific recognition of *cis*-acting promoter elements by *trans*-acting sequence-specific DNA binding transcription factors (TFs) (Kim and Zhang, 2004). Among the several classes of TFs, the DNA-binding proteins containing WRKY domains have been shown to be associated with plant defense responses (Pandey and Somssich, 2009; Tripathi et al., 2014).

WRKY TFs are one of the largest families of transcriptional regulators in plants and are characterized by the presence of one or two 60-amino-acid WRKY domains (Rushton et al., 2010). A common feature of the WRKY domain is the highly conserved WRKYGQK sequence at its N-terminus along with a zinc-finger binding motif at its C-terminus. It is generally assumed that the WRKY domain can activate or repress the transcription of target genes by specific binding to various W-box elements with an invariant GAC core sequence present in the promoters (Brand et al., 2013a,b). In addition, based on the number of WRKY domains and the features of the zinc-finger motifs, WRKY proteins can be divided into three groups: group I contains two WRKY domains with a C2H2 zinc-finger motif; group II has one WRKY domain and a C2H2 zinc-finger motif; and group III contains one WRKY domain and a different C2HC zinc-finger motif (Eulgem et al., 2000).

To date, many group III WRKY TFs have been identified in *Arabidopsis*, rice, Pak-choi, *Thlaspi caerulescens*, and *Vitis pseudoreticulata* (Kalde et al., 2003; Xie et al., 2005; Wei et al., 2008; Li et al., 2010; Wang et al., 2012). Additionally, more group III WRKY TFs have been reported to be involved in plant responses to abiotic stress. For instance, *BcWRKY46* overexpression in tobacco enhanced the tolerance of transgenic tobacco to drought, cold, and salt stress (Wang et al., 2012). FcWRKY70, a WRKY protein of *Fortunella crassifolia*, functions in drought tolerance and modulates putrescine synthesis by regulating the arginine decarboxylase gene (Gong et al., 2015). WRKY70 and WRKY54 co-operate as negative regulators of stomatal closure and, consequently, osmotic stress tolerance in *Arabidopsis* (Li et al., 2013). Another group III WRKY TF, *AtWRKY46*, has been shown to play dual roles in regulating plant responses to drought and salt stress and light-dependent stomatal opening in guard cells (Ding et al., 2014). Furthermore, the phytohormone abscisic acid (ABA) plays central role in the stress responses of plants exposed to environmental challenges. A recent study has demonstrated that WRKY TFs constitute key nodes in ABA-responsive signaling networks (Rushton et al., 2012). Several rice WRKY proteins have been found to act as repressors or activators of an ABA-inducible promoter in aleuronic cells (Xie et al., 2005). Ren et al. (2010) also showed that *AtWRKY63* can bind the W-box in the promoter of *AREB1*/*ABF2 in vitro*, and the *wrky63* mutant is more sensitive to drought stress than wild-type plants.

In addition, emerging evidence has indicated that group III WRKY TFs are central components of many aspects of the plant innate immune system, including basal defense and systemic-acquired resistance (Rushton et al., 2010; Jiang et al., 2014). The WRKY TFs bind to and regulate the expression of several well-characterized plant defense-related genes, all of which contain W-box elements in their promoter regions (Yu et al., 2001). For example, Mao et al. (2007) reported that WRKY62 acts downstream of cytosolic NPR1 and negatively regulates JA-responsive gene expression in *Arabidopsis*. *Arabidopsis* WRKY70 has been shown to modulate the crosstalk between SA- and JA-mediated signaling by promoting SA-dependent and suppressing JA-dependent responses (Li et al., 2006). In rice plants, overexpression of the elicitor-induced *OsWRKY53* gene leads to enhanced resistance to the blast fungus *Magnaporthe grisea* (Chujo et al., 2007). OsWRKY31 acts as a transcriptional activator, and overexpression of the *OsWRKY31* gene enhances resistance against infection with *M. grisea* (Zhang et al., 2008). A pepper (*Capsicum annuum* L.) WRKY gene, *CaWRKY30*, is involved in pathogen stress responses (Zheng et al., 2011). Another group III member, AtWRKY52/RRS1, forms a receptor complex by combining with several NB-LRR proteins, and this receptor complex integrates a “decoy” domain that enables the detection of effectors that target WRKY proteins (Sarris et al., 2015). These findings further emphasize the significance of group III WRKY proteins for plant immunity.

Cotton (*Gossypium hirsutum*) is an important fiber and oil crop around the world, and its growth and yield are affected by various biotic and abiotic stress conditions. Previous studies have primarily focused on group I and II WRKY TFs in cotton. For example, a group I WRKY TF from cotton, *GhWRKY3*, was shown to be responsive to biotic stresses and various phytohormones (Guo et al., 2010). The group II WRKY TF, *GhWRKY17* responds to drought and salt stress through ABA signaling and the regulation of cellular reactive oxygen species (ROS) production (Yan et al., 2014), and *GhWRKY40* overexpression in tobacco results in enhanced susceptibility to biotrophic pathogen infections (Wang et al., 2014). However, few group III WRKY TFs have been functionally characterized in cotton. Here, we report the identification and functional characterization of a group III WRKY transcription factor, *GhWRKY27a*. The expression of *GhWRKY27a* was induced by various abiotic and biotic stresses. The silencing of *GhWRKY27a* enhanced the tolerance of cotton plantlets to drought stress. In addition, ectopic expression of *GhWRKY27a* in *Nicotiana benthamiana* led to enhanced susceptibility to drought stress and infection by the fungal pathogen *Rhizoctonia solani*. This study provides key clues toward understanding the roles of *GhWRKY27a* in plant defense responses to biotic and abiotic stresses.

## Materials and methods

### Plant growth and various treatments

Cotton (*G. hirsutum* L. cv. lumian 22) seedlings were grown in an environmentally controlled growth chamber at 26 ± 1°C with a 16 h light/8 h dark cycle (relative humidity of 60–75%). Seven-day-old cotton seedlings were collected for various treatments. For the temperature treatment, uniformly developed cotton seedlings were transferred to cold conditions (4°C) for indicated time periods. For other treatments, uniformly developed seedlings were cultured or sprayed with NaCl (200 mM), 15% poly(ethylene glycol) 6000 (w/v), H_2_O_2_ (10 mM), ABA (100 μM), MeJA (100 μM), SA (2 mM), or ET released from the ethephon (5 mM), or were wounded. For the fungal pathogen treatment, the roots of cotton seedlings were dipped into *R. solani* conidial suspensions (10^5^ conidia mL^−1^). The treated cotyledons were collected for RNA extraction. Additionally, *N. benthamiana* seeds were surface sterilized and planted on Murashige and Skoog (MS) medium for germination under greenhouse conditions. *N. benthamiana* seedlings at the two- or three-leaf stage were transplanted into soil and maintained under a 16 h light/8 h dark photoperiod at 25°C. The resulting uniform seedlings were used for further study. Each treatment was performed at least three times.

### Cloning of *GhWRKY27a*

The full-length cDNA and genomic sequence of *GhWRKY27a* were obtained as previously described (Yu et al., 2012). The general PCR procedures and primers are shown in Tables S1, S2. Multiple protein sequence alignments amongst homologs were conducted using DNAman 6.0.3 software and the NCBI bioinformatics tools (http://blast.ncbi.nlm.nih.gov/Blast.cgi). Phylogenetic analysis was performed using Molecular Evolutionary Genetics Analysis (MEGA version 5.1) software using the neighbor-joining method.

### Subcellular localization of *GhWRKY27a*

The coding region of the *GhWRKY27a* gene without the stop codon was inserted at the 5′-terminal end of the GFP gene to generate pBI121-GhWRKY27a-GFP, which is driven by the *Cauliflower mosaic virus* 35S (CaMV35S) promoter. The *Agrobacterium tumefaciens* strain GV3101 carrying the pBI121-GhWRKY27a-GFP fusion construct or the positive control pBI121-GFP construct was inoculated into fully expanded leaves of 6-week-old *N. benthamiana*. The lower epidermis cells were analyzed using an LSM 510 confocal laser-scanning microscope (Carl Zeiss, Germany) operated with LSM Image Browser software.

### Virus-induced gene silencing (VIGS) of *GhWRKY27a* in cotton

For VIGS silencing of *GhWRKY27a*, the tobacco rattle virus (TRV)-based VIGS system was employed. A 481-bp fragment was inserted into the multiple cloning site in plasmid pTRV-RNA2 to produce pTRV-RNA2-GhWRKY27a. *A. tumefaciens* strain GV3101 carrying pTRV-RNA2-GhWRKY27a, the pTRV-RNA2-GhCLA fusion construct or the pTRV-RNA2 construct was combined with the pTRV-RNA1 strain (1:1 ratio; OD_600_ = 1.0) and co-infiltrated into two fully expanded cotyledons of cotton as described by Dang et al. (2013).

### Vector construction and plant transformation

Under the control of the CaMV35S promoter, the *GhWRKY27a* ORF was cloned into the *Xba* I/*Sal* I sites of the binary vector pBI121. The recombinant plasmid was then introduced into *A. tumefaciens* (strain LBA4404) for *N. benthamiana* transformation using the leaf disc method, and transformants were screened for kanamycin (100 mg L^−1^) resistance and further confirmed by PCR. The transgenic T_3_ lines were used in experiments. All of the primers used in this study are listed in Table S1.

### Quantification of endogenous ABA content

Samples were homogenized in liquid nitrogen and extracted in ice-cold phosphate-buffered saline (PBS, pH 7.4). After centrifugation at 4000 g for 20 min, the supernatant was dried in N_2_ and subsequently dissolved for ELISA assay using a kit (Fangcheng, Beijing, China) according to the manufacturer's instructions.

### 3,3′-diaminobenzidine (DAB) and nitro blue tetrazolium (NBT) staining assays

For DAB staining, *N. benthamiana* and cotton leaves were incubated in DAB solution (1 mg mL^−1^, pH 3.8) for 15 h at 25°C in the dark. After staining, the leaves were soaked in 95% ethanol overnight to remove chlorophyll. For the NBT assays, leaves were incubated in NBT solution (0.1 mg mL^−1^) for 15 h at 25°C in the dark. After staining, leaves were soaked in 95% ethanol overnight to remove chlorophyll.

### Oxidative stress experiments

For oxidative stress analysis, uniform leaf discs were detached from healthy and fully expanded wild type and transgenic plants and floated in 12 mL of a solution containing one of three concentrations of methyl viologen (MV) (0, 400, or 600 μM) for 72 h. Subsequently, the chlorophyll contents were extracted using 95% ethanol and analyzed using spectrophotometry.

### Pathogen inoculation and disease resistance test

Leaves of 7-week-old transgenic and wild-type plants were inoculated with *R. solani* spore suspensions (10^5^ conidia mL^−1^) prepared in 1% glucose. Inoculated leaves were kept in a transparent box under greenhouse conditions. Lesions were measured at 7 days after inoculation. Furthermore, infection was confirmed by inoculation with the above suspensions of *R. solani* spores using the trickle irrigation method (Li et al., 2014a).

### RNA extraction and quantitative PCR

Total RNA was isolated from samples using the modified cetyltrimethylammonium bromide (CTAB) method (Lu et al., 2013) or TRIzol reagent (TaKaRa, Dalian, China). Next, the RNA was used to obtain first-strand cDNA using the EasyScript First-strand cDNA Synthesis SuperMix kit (TransGen Biotech, Beijing, China) according to the manufacturer's instructions. Real-time quantitative PCR (qRT-PCR) was carried out using the SYBR® PrimeScript™ RT-PCR Kit (TaKaRa, Dalian, China) and a CFX96TM Real-time System (Bio-Rad, Hercules, CA, USA) following the procedures described by Shi et al. (2014). The primers used in the qRT-PCR analyses are shown in Table S3. The *G. hirsutum* ubiquitin (*UBI*) and *N. benthamiana* β*-actin* genes were used as internal controls. Data were analyzed using the CFX Manager software, version 1.1, and significant differences were identified using Duncan's multiple range tests with Statistical Analysis System (SAS) software, version 9.1. All reactions were performed with three technical replicates.

## Results

### Sequence analysis of GhWRKY27a

The full-length cDNA of the *GhWRKY27a* (GenBank accession number: KM453243) sequence consisted of 1513 nucleotides, including a 1068-bp open reading frame (ORF), a 319-bp 5′-untranslated region (5′-UTR) and a 126-bp 3′-UTR. The ORF encoded a protein composed of 356 amino acid residues with a predicted molecular mass and isoelectric point of 40.062 kDa and 5.46, respectively.

**Figure 1:**
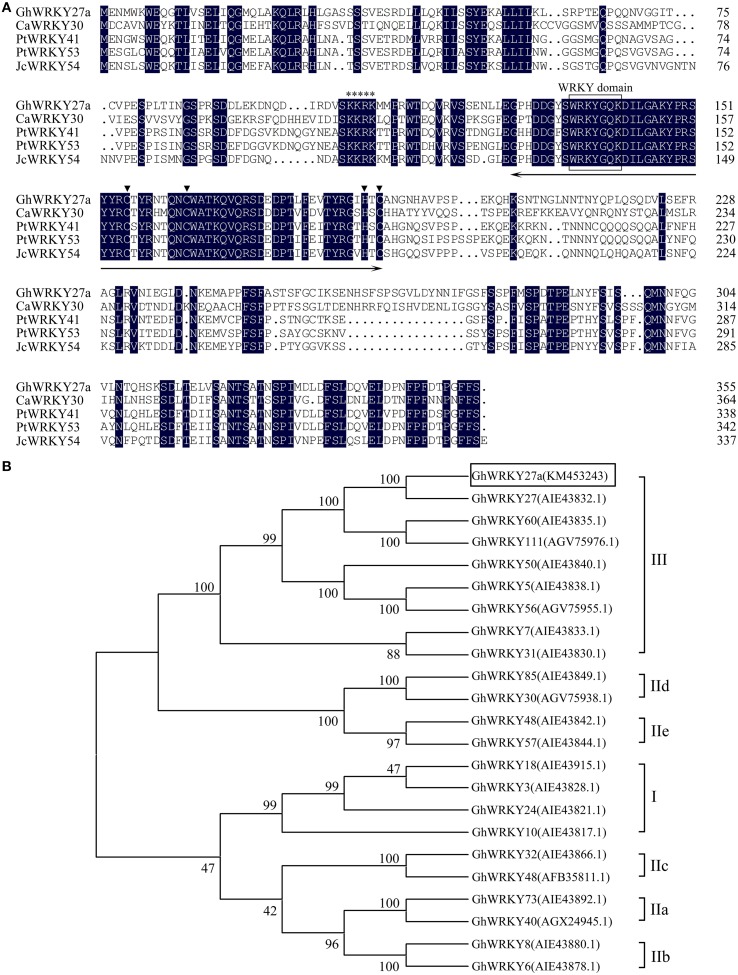
**Sequence analysis of GhWRKY27a**. **(A)** Alignment of the amino acid sequence of GhWRKY27a with the sequences of CaWRKY30, PtWRKY41, PtWRKY53, and JcWRKY54. Identical amino acids are shaded in black. The approximately 60-amino acid WRKY domain and the C and H residues in the zinc-finger motif (C-X_7_-C-X_23_-H-X_1_-C) are indicated with a two-headed arrow and inverted triangle, respectively. The highly conserved amino acid sequence WRKYGQK in the WRKY domain is boxed. The putative nuclear localization signal, KKRK, is indicated with an asterisk. **(B)** Phylogenetic analysis of GhWRKY27a in relation to other cotton WRKY TFs. A neighbor-joining phylogenetic tree was created using MEGA 5.1 software. GhWRKY27a is highlighted in the box, and each gene name is followed by its protein ID.

The deduced amino acid sequence of GhWRKY27a was closely related to those of *C. annuum* CaWRKY30 (GenBank accession number: ACJ04728.1, 48.49% protein sequence identity), *Populus trichocarpa* PtWRKY41 (GenBank accession number: XP_002297983.1, 66.11% protein sequence identity), *P. trichocarpa* PtWRKY53 (GenBank accession number: XP_002304549.1, 64.46% protein sequence identity), and *Jatropha curcas* JcWRKY54 (GenBank accession number: AGQ04248.1, 61.71% protein sequence identity). The WRKY domain and the C and H residues in the zinc-finger motif (C-X_7_-C-X_23_-H-X_1_-C) were identified, indicating that GhWRKY27a belongs to group III of the WRKY family. Additionally, a putative nuclear localization signal (NLS), KKRK, was found at position 105–108 (Figure 1A).

To investigate the evolutionary relationship of the cloned WRKY protein to other known cotton WRKYs, a neighbor-joining analysis was performed with the obtained amino acid sequences. As shown in Figure 1B, GhWRKY27a was highly similar to group III WRKY family members, which is consistent with the results of the amino acid alignment analysis. These results strongly imply that GhWRKY27a is a member of WRKY group III.

To further elucidate the properties of *GhWRKY27a*, the genomic DNA sequence of *GhWRKY27a* (GenBank accession number: KM453244), which consisted of 1696 bp (containing three exons and two introns), was obtained. Comparative analysis of *GhWRKY27a* and the other group III WRKY TF genomic sequences revealed that the numbers and positions of the introns in these genes were highly conserved (Figure S1).

### Ghwrky27a is localized to the nucleus

**Figure 2:**
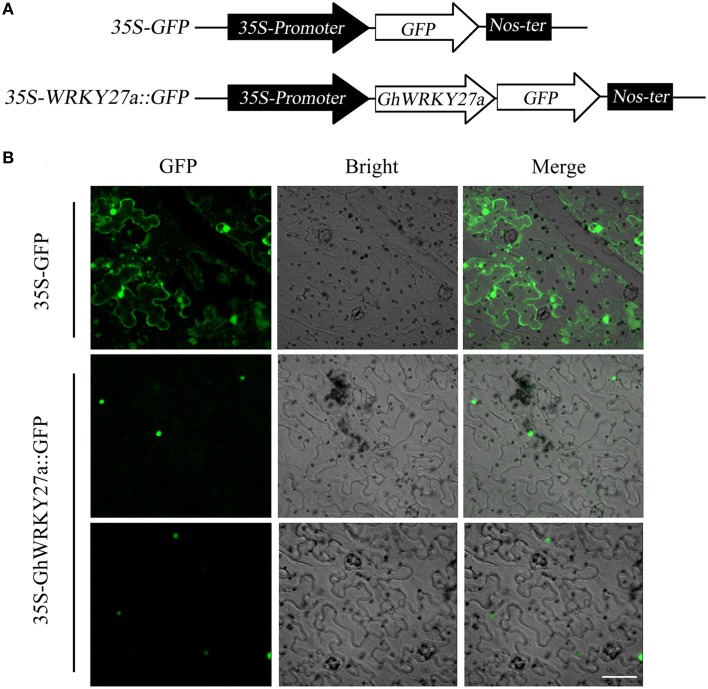
**Subcellular localization of the GhWRKY27a protein transiently expressed in *N. benthamiana* cells**. **(A)** Schematic representation of the 35S-GhWRKY27a::GFP fusion construct and the control 35S-GFP construct. **(B)** The transient expression of 35S-GhWRKY27a::GFP in *N. benthamiana* cells was examined at 48 h after transformation under a Zeiss LSM 510 confocal laser-scanning microscope. The scale bar represents 50 μm.

Bioinformatics analysis using the PSORT program predicted that GhWRKY27a would localize to the nucleus. To confirm this prediction, the *GhWRKY27a* ORF was fused in-frame to the green fluorescent protein (GFP) gene under the control of the cauliflower mosaic virus CaMV35S promoter (Figure 2A). As shown in Figure 2B, typical results indicated exclusive localization of GhWRKY27a-GFP to the nucleus in *N. benthamiana* epidermal cells, whereas GFP alone localized to multiple subcellular compartments, including the cytoplasm and nucleus. These results indicated that the GhWRKY27a protein localized to the nucleus.

### Expression profiles of *GhWRKY27a* under stress conditions

**Figure 3:**
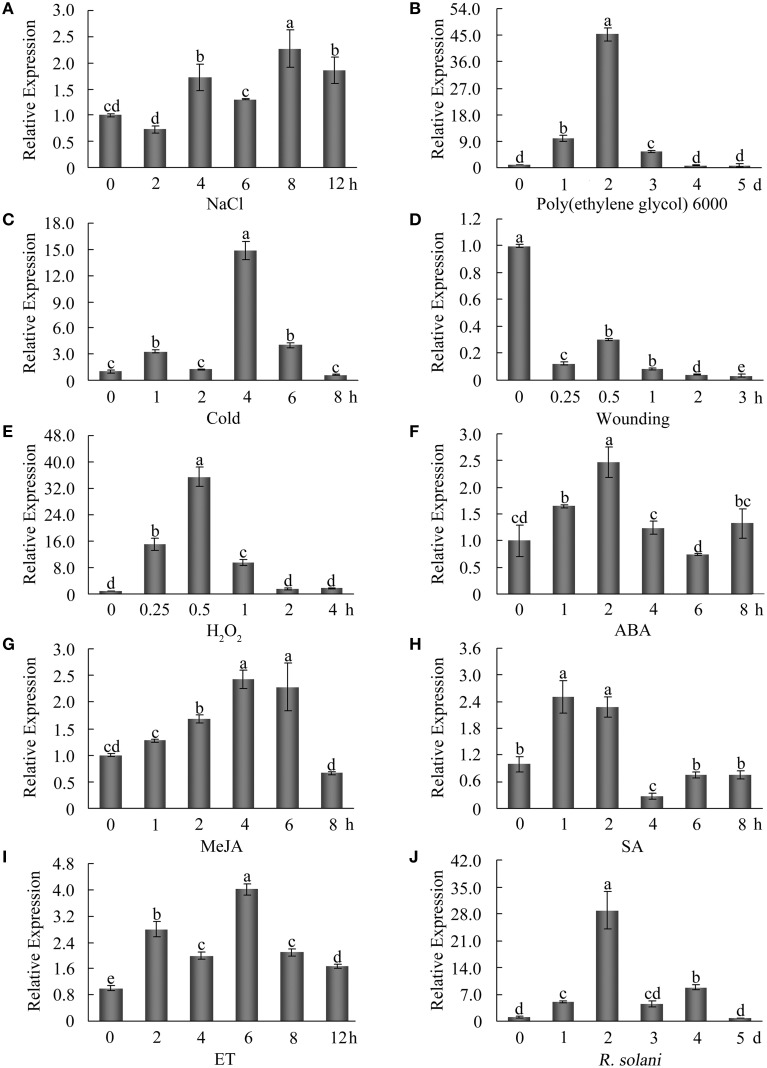
**Expression profiles of *GhWRKY27a* under diverse stress conditions**. Seven-day-old cotton seedlings in hydroponic culture were subjected to the following treatments: 200 mM NaCl **(A)**, 15% poly(ethylene glycol) 6000 **(B)**, cold **(C)**, wounding **(D)**, 10 mM H_2_O_2_ **(E)**, 100 μM ABA **(F)**, 100 μM MeJA **(G)**, 2 mM SA **(H)**, 5 mM ET released from the ethephon **(I)**, and *Rhizoctonia solani* **(J)**. Total RNA was isolated at the indicated times after treatment and subjected to qRT-PCR analysis. The *ubiquitin* gene (GenBank accession number: EU304080) was employed as an internal control. The data are the mean ± SE of three independent experiments. The letters above the columns represent significant differences (*P* < 0.01) based on Duncan's multiple range test.

To examine the expression patterns of *GhWRKY27a* following various environmental stresses, 7-day-old cotton seedlings were exposed to various stresses. As shown in Figure 3A, NaCl treatment induced a slight increase in *GhWRKY27a* expression. Following poly(ethylene glycol) 6000 and cold treatments, *GhWRKY27a* transcription was dramatically elevated, peaking after 2 days and 4 h, respectively (Figures 3B,C). Conversely, the expression of *GhWRKY27a* was downregulated after wounding treatment (Figure 3D). In addition, to elucidate *GhWRKY27a*-related signal transduction mechanisms, we also examined the responsiveness of *GhWRKY27a* to diverse signaling molecules. As shown in Figures 3E–I, the expression of *GhWRKY27a* was notably increased at different time points by H_2_O_2_, ABA, methyl jasmonate (MeJA), salicylic acid (SA) and ethylene (ET) treatments, and it then decreased markedly. Moreover, the fungal pathogen *R. solani* increased *GhWRKY27a* transcript levels (Figure 3J). These results indicate that *GhWRKY27a* may be involved in responses to multiple abiotic and biotic stresses by mediating multiple plant defense signal transduction pathways.

### Silencing *GhWRKY27a* enhanced drought tolerance in cotton

**Figure 4:**
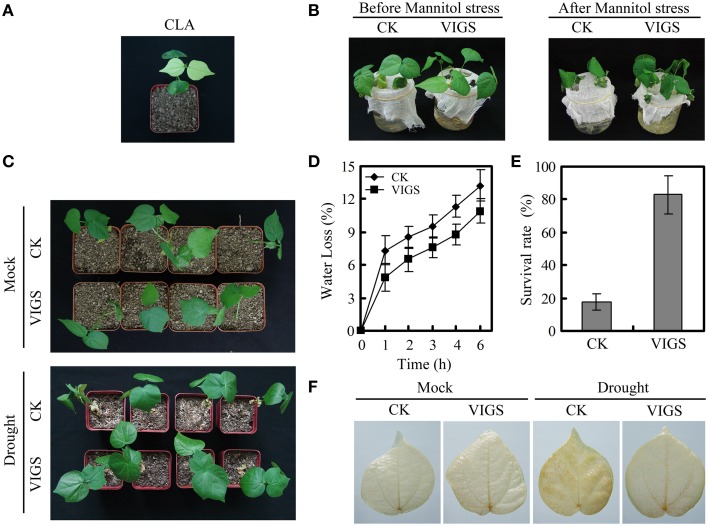
**Silencing *GhWRKY27a* enhanced drought tolerance in cotton**. **(A)** Silencing of cotton CLA was phenotypically visible at 20 days after agroinfiltration. **(B)** Phenotype of mannitol-stressed plants. The plants were soaked with 400 mM mannitol solution for 12 h approximately 3 weeks post-inoculation. **(C)** Phenotype of drought-stressed plants. Water was withheld from CK and VIGS plants for 7 days. **(D)** Water loss from detached leaves of CK and VIGS plants. The rate of water loss was calculated based on the decrease in the fresh weight of the samples. The data are presented as the mean ± standard error of three independent experiments. **(E)** Survival rates of CK and VIGS plants under drought stress. The data are presented as the mean ± standard error of three independent experiments. **(F)** Drought-induced H_2_O_2_ as detected via DAB staining.

To evaluate the role of *GhWRKY27a* in the drought stress response, we employed a VIGS technique to knock down the expression of *GhWRKY27a* in cotton. The cotton *CLA* gene was used as an additional control to determine the efficiency of gene silencing (Figure 4A). The transcript levels of *GhWRKY27a* in *GhWRKY27a*-silenced (VIGS) and empty vector-treated cotton (CK) plants were analyzed via qRT-PCR. The downregulation of *GhWRKY27a* indicated that *GhWRKY27a* was successfully knocked down in the VIGS plants (Figure S2). As shown in Figure 4B, we did not observe any difference in morphology and growth between VIGS and CK plants. However, after mannitol treatment, the CK plants exhibited severe wilting compared with the VIGS plants (Figure 4B). Likewise, after 7 days of water-withholding treatment, the CK plants began wilting, while the VIGS plants were less affected (Figure 4C). In addition, the VIGS plants exhibited less water loss and a higher survival rate than CK plants (Figures 4D,E). Moreover, less H_2_O_2_ accumulation was observed via DAB staining in the VIGS plants after drought treatment (Figure 4F). These data above indicated that silencing of *GhWRKY27a* enhanced drought tolerance in cotton.

### *GhWRKY27a* overexpression decreased tolerance to mannitol treatments during seed germination and root elongation

To further confirm the function of *GhWRKY27a*, transgenic *N. benthamiana* plants overexpressing *GhWRKY27a* were generated. Eight independent transgenic lines were selected on kanamycin and the chromosomal integration of the transgene was confirmed through PCR detection using genomic DNA as a template (Figure S3). Three bona fide GhWRKY27a expressing transgenic lines (OE1, OE2, and OE3) were chosen randomly, and the T_3_ transgenic plants were used for further experiments. The wild-type (WT) plants were germinated at the same time with the transgenic plants.

**Figure 5:**
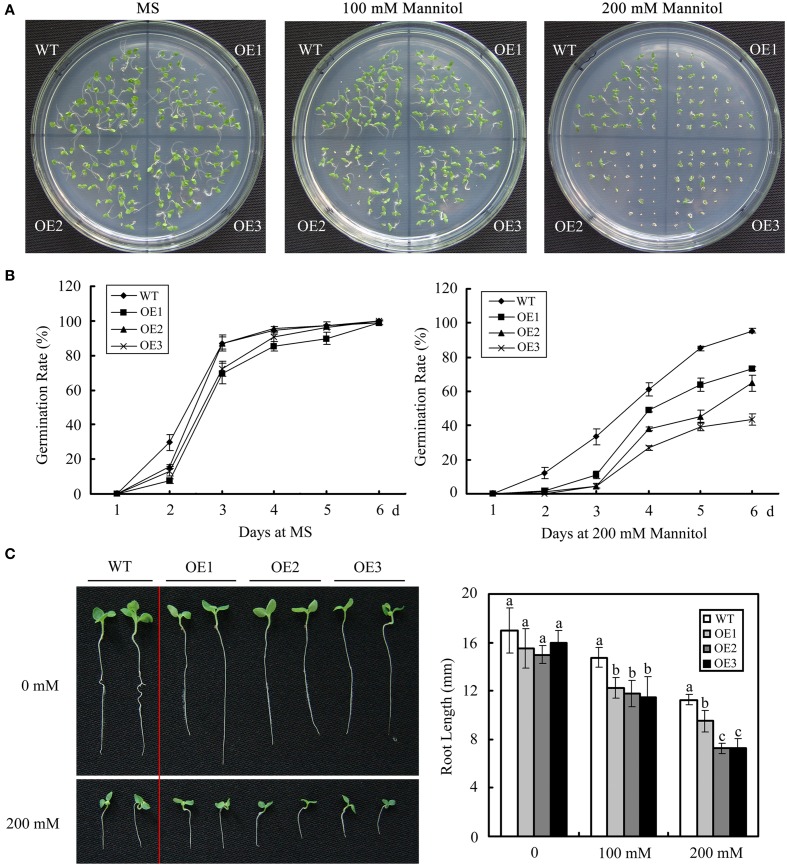
***GhWRKY27a* overexpression enhances osmotic sensitivity during seed germination**. **(A)** Seed germination on MS medium with 0, 100, and 200 mM mannitol. **(B)** Germination rates of the WT and OE lines under normal and 200 mM mannitol treatment conditions. Germination was scored daily. The data are the means ± SE of three independent experiments. **(C)** Photographs and measurements of the root lengths of WT and OE seedlings supplemented with mannitol. The data are the means ± SE of three independent experiments. The letters above the columns represent significant differences (*P* < 0.05) based on Duncan's multiple range test.

The potential effects of *GhWRKY27a* on osmotic stress were investigated by comparing *GhWRKY27a*-overexpressing (OE) plants with WT plants grown on 1/2 MS medium with or without mannitol. In the absence of mannitol, as shown in Figures 5A,B, no significant difference in seed germination rate was observed between WT and OE plants. However, in the presence of mannitol, OE plants showed enhanced sensitivity to mannitol-induced osmotic stress. The germination of OE seeds was more severely inhibited than that of WT lines. We next tested whether *GhWRKY27a* influences the growth of post-germinated tobacco seedlings under mannitol stress. At 3 days after sowing on 1/2 MS medium, seeds from the WT and OE lines showing radicle emergence were transferred to medium containing various mannitol concentrations ranging from 0 to 200 mM. The root length was observed to be shorter in all of the OE plants compared with WT plants in the presence of mannitol (Figure 5C). These data show that *GhWRKY27a* overexpression enhances osmotic sensitivity during seed germination.

### *GhWRKY27a* overexpression reduced tolerance to drought stress in transgenic plants

**Figure 6:**
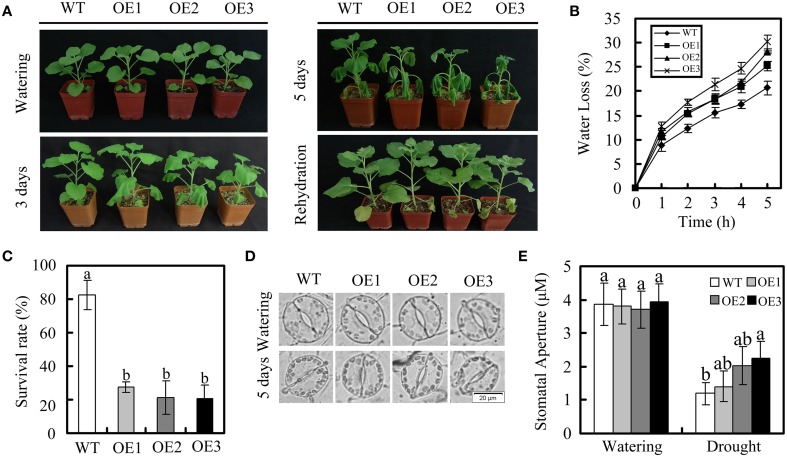
**Dehydration response of *GhWRKY27a*-overexpressing plants**. **(A)** The WT and OE plants were grown until they produced eight expanded leaves after which irrigation was stopped to induce dehydration. Representative plants exposed to 3 or 5 days of drought are shown. After 1 week of drought stress, plants were rehydrated, and their recovery was recorded. **(B)** Water loss from detached leaves of WT and OE plants. The rate of water loss was calculated based on the decreases in the fresh weights of the samples. **(C)** Survival rates of WT and OE plants under drought stress. **(D,E)** Stomatal changes were observed with a microscope before and after drought treatment. The data are presented as the mean ± standard error of three independent experiments. The values indicated by the different letters are significantly different at *P* < 0.05, as determined using Duncan's multiple range test.

We next tested the phenotypes of the transgenic plants under drought stress. WT and OE plants at the vegetative growth stage were stopped to induce dehydration. After 3 days, the non-irrigated OE plants started to wilt, but the non-irrigated WT plants were still turgid (Figure 6A). The WT plants started to show wilting symptoms 2 days later. After 1 week of drought treatment, the plants were irrigated again. The WT plants recovered, while several of the OE leaves did not recover completely. In a further study, leaf water loss, the survival rate, and the stomatal aperture were compared in OE plants and WT plants after drought treatment. As shown in Figure 6B, the rate of water loss was higher in the OE plants than in WT plants. Moreover, the survival rate of the OE plants was lower than that of the WT plants (Figure 6C). The stomatal aperture of OE plants did not differ significantly from that of WT plants under normal conditions. However, the degree of stomatal closure observed in WT plants was greater than in OE plants under drought conditions (Figure 6D).

**Figure 7:**
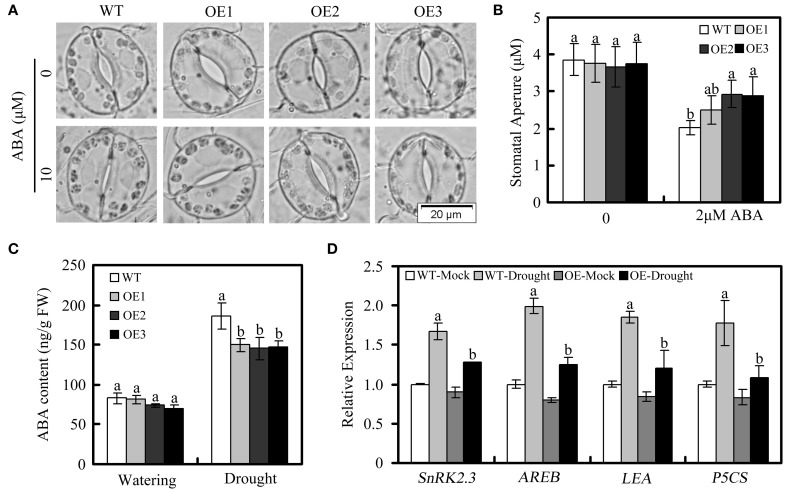
**Stomatal behavior in response to ABA and relative expression of ABA- and drought-responsive genes in WT and OE plants under drought treatment**. **(A,B)** Stomatal closure in response to ABA treatment. **(C)** Endogenous ABA levels in WT and OE plants under normal and drought conditions. **(D)** Expression of ABA- and drought-responsive genes, determined via qRT-PCR. The *actin* gene was used to normalize the amount of template in each reaction. The transcript levels of the respective genes in mock-treated wild-type plants were used as a reference and set to a value of “1.” The data are presented as the mean ± standard error of three independent experiments. The values indicated by the different letters are significantly different at *P* < 0.05, as determined using Duncan's multiple range test.

To further investigate the *GhWRKY27a*-associated mechanisms resulting in drought sensitivity, we measured the stomatal aperture in OE plants and WT plants in response to ABA because the ABA responsiveness of stomatal movement can modify drought sensitivity (Blatt, 2000). In the absence of ABA, there were no obvious differences in stomatal aperture between WT and OE plants. Following treatment with 10 μM ABA for 3 h, the degree of stomatal closure was greater in WT plants than in OE plants (Figures 7A,B). In addition, we found that the endogenous ABA levels increased to a greater extent in WT plants than in OE plants under drought treatment, which is consistent with the higher degree of stomatal closure observed in the WT plants (Figure 7C). However, in the absence of the stress treatment, the ABA contents of WT and OE plants were not significantly different (Figure 7C). Furthermore, many studies have shown correlations between drought sensitivity and the expression of ABA- or drought-related genes (Yan et al., 2014; Jia et al., 2015). In this study, *NbSnRK2.3* (sucrose non-fermenting 1-related protein kinase), *NbAREB1* (ABA-responsive element binding), *NbLEA* (late embryogenesis abundant), and *NbP5CS* (delta1-pyrroline-5-carboxylate synthetase) were used to monitor ABA and drought stress responses in OE plants. Under drought conditions, the levels of these genes in OE plants were lower than in WT plants (Figure 7D). Taken together, these data indicate that the drought-sensitive phenotype of OE plants is associated with enhanced stomatal opening and lower levels of ABA- or drought-related gene expression.

### *GhWRKY27a* overexpression in transgenic plants reduced ROS scavenging ability under drought stress

**Figure 8:**
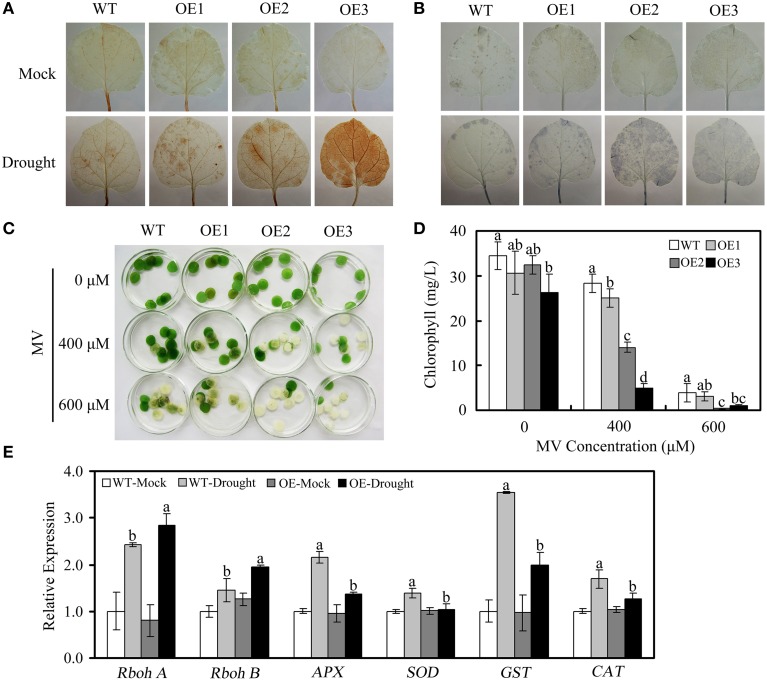
**ROS accumulation and expression of ROS-related genes in WT and OE plants under drought conditions**. **(A,B)** Drought-induced H_2_O_2_ and ${\text{O}}_{2}^{-}$ accumulation, as detected via DAB staining and NBT staining, respectively. **(C)** Phenotypes of leaf disks from WT and OE plants that were incubated in different concentrations of MV (0, 400, or 600 μM). **(D)** Relative chlorophyll content in the leaf disks from **(C)**. **(E)** Expression of ROS-related genes, determined via qRT-PCR. The *actin* gene was used to normalize the amount of template in each reaction. The transcript levels of the respective genes in mock-treated wild-type plants were used as references and set to a value of “1.” The data are presented as the mean ± standard error of three independent experiments. The values indicated by the different letters are significantly different at *P* < 0.05, as determined using Duncan's multiple range test.

Abiotic stress results in the accumulation of ROS in plants. Therefore, we evaluated the accumulation of H_2_O_2_ and superoxide radical anions (${\text{O}}_{2}^{-}$) in the leaves of WT and OE plants under drought stress. Leaves detached from untreated WT and OE plants were used as controls. As shown in Figures 8A,B, under drought stress, OE plants showed greater accumulation of H_2_O_2_ and ${\text{O}}_{2}^{-}$ than WT plants, as indicated by the accumulation of brown (DAB staining) and blue (NBT staining) pigments. Under normal growth conditions, no obvious differences in H_2_O_2_ or ${\text{O}}_{2}^{-}$ were detected in WT vs. OE plants.

To confirm the ability of *GhWRKY27a*-overexpressing plants to scavenge ROS, the oxidative agent MV was used. As shown in Figure 8C, cotyledon bleaching or chlorosis was more severe in the OE plants than in the WT plants. This result was confirmed by measuring chlorophyll content after MV treatment. The WT plants demonstrated higher chlorophyll content than the OE plants (Figure 8D), suggesting that *GhWRKY27a* overexpression conferred decreased tolerance to oxidative stress.

To ascertain the possible mechanisms underlying the reduced antioxidant defense abilities observed in the transgenic plants, the levels of defense-related genes were assessed in WT and OE plants via qRT-PCR under drought stress. As shown in Figure 8E, the levels of superoxide dismutase gene (*SOD*), glutathione S-transferase gene (*GST*), ascorbate peroxidase gene (*APX*), and catalase gene (*CAT*), which encode ROS-scavenging enzymes, were increased to a greater extent in WT plants than in OE plants under drought conditions. However, the levels of the ROS producers (the respiratory burst oxidase homolog genes *RbohA* and *RbohB*) were increased to a greater extent in OE plants than in WT plants (Figure 8E). Together, these data demonstrate that *GhWRKY27a* may play a critical role in the regulation of the ROS network pathway.

### *GhWRKY27a* overexpression enhanced susceptibility to *R. solani*

**Figure 9:**
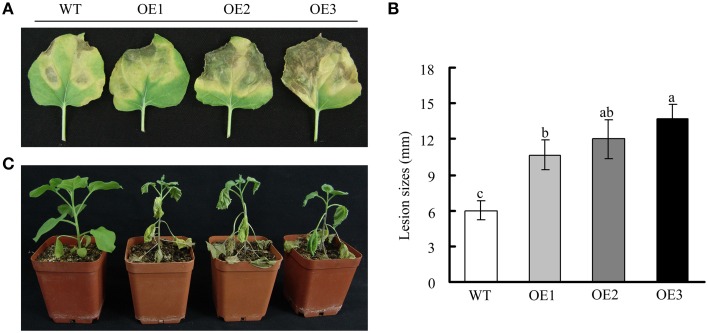
**Effects of constitutive *GhWRKY27a* overexpression on susceptibility to *R. solani***. **(A)** Disease symptoms in WT and OE plants at 7 days after inoculation with *R. solani*. **(B)** The lesion sizes induced by *R. solani* infection were assessed. Different letters above the columns indicate significant differences (*P* < 0.001) according to Duncan's multiple range test. **(C)** Symptoms of transgenic plants inoculated with *R. solani* using the trickle irrigation method.

The upregulation of *GhWRKY27a* expression observed in response to *R. solani* inoculation and exogenous SA, ET, and MeJA application suggested a role for this gene in plant immunity. To investigate the role of *GhWRKY27a* in disease resistance in plants, detached leaves from 2-month-old T_3_ generation transgenic plants were incubated with *R. solani*, which is a necrotrophic pathogen. As shown in Figures 9A,B, spreading necrosis and more severe disease symptoms were observed in OE plants. In contrast, WT plants were essentially resistant and exhibited only non-spreading local necrosis lesions at the inoculation sites. When the plants were infected via the trickle irrigation method for approximately 10 days, caudex rot was observed to be more serious in OE plants than in WT plants (Figure 9C).

**Figure 10:**
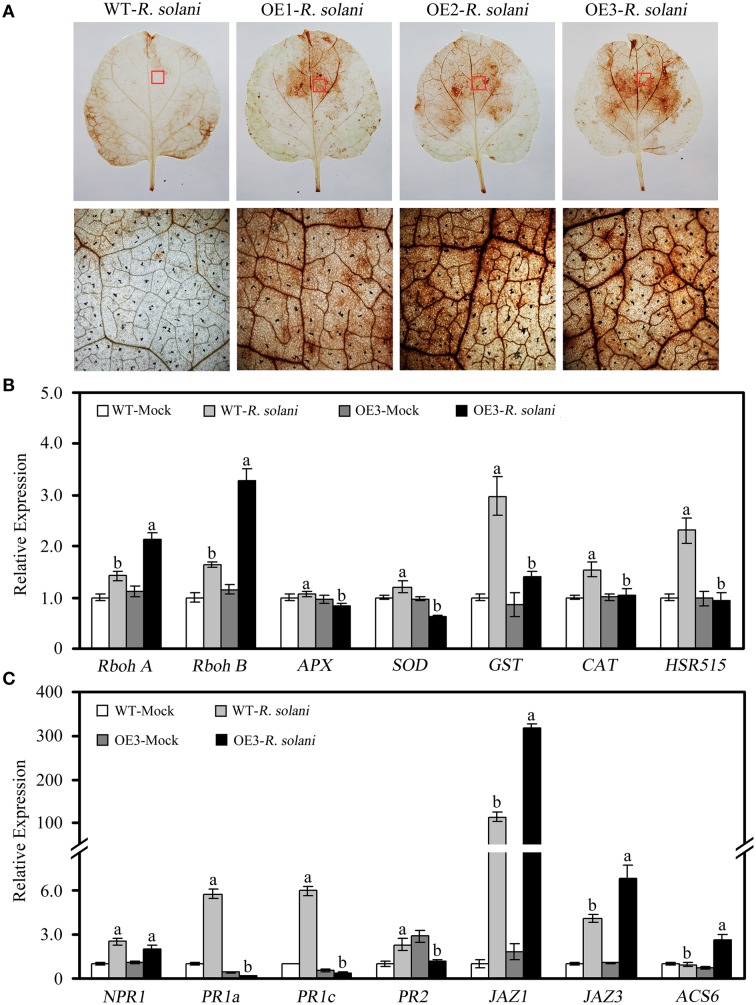
**The accumulation of H_2_O_2_ and relative expression of defense-related genes following *R. solani* infections**. Leaf samples were collected at 7 days after inoculation with *R. solani*. **(A)** Accumulation of H_2_O_2_. **(B)** Relative expression of ROS production-related genes and HR-associated genes. **(C)** Relative expression of genes involved in different defense signaling pathways. The *actin* gene was used to normalize the amount of template in each reaction. The transcript levels of the respective genes in mock-treated wild-type plants were used as a reference and set to a value of “1.” The data are presented as the mean ± standard error of three independent experiments. The values indicated by different letters are significantly different at *P* < 0.05, as determined using Duncan's multiple range test.

To further elucidate possible mechanisms of *GhWRKY27a*-mediated disease sensitivity, we examined the effects of overexpressing *GhWRKY27a* on the accumulation of H_2_O_2_ and the transcript levels of defense genes following *R. solani* infection. As shown in Figure 10A, histochemical staining with DAB revealed that the *in situ* accumulation of H_2_O_2_ in OE leaves was higher than in the leaves of WT plants after *R. solani* infection. In addition, compared with WT plants, the expression levels of *Rboh A* and *Rboh B*, which encode ROS-generating enzymes, was increased to a greater extent in OE plants (Figure 10B). The levels of *APX, SOD, GST*, and *CAT*, which are involved in the scavenging of ROS, were repressed in the OE plants, as shown in Figure 10B. Likewise, compared with WT plants, the levels of the pathogenesis-related (PR) genes *PR1a* and *PR1c*, which are thought to be regulated by the SA-mediated signaling pathway (Dang et al., 2013), as well as the hypersensitivity-related (HSR) gene *HSR515*, which is considered to be associated with the hypersensitive response (Dang et al., 2013), were decreased significantly in OE plants following *R. solani* infection (Figures 10B,C). However, the levels of *JAZ1* and *JAZ3*, which are known to be associated with the JA signaling pathway (Pauwels and Goossens, 2011), were increased in OE plants compared with WT plants (Figure 10C). Similarly, the transcript levels of the ET-responsive gene *ACS6* were increased (Figure 10C). Furthermore, no significant differences in the levels of *nonexpresser of PR genes 1* (*NPR1*) was observed between WT and OE plants (Figure 10C). These results suggest that the enhanced susceptibility of *GhWRKY27a*-overexpressing plants to *R. solani* infection is associated with the altered expression of defense-related genes.

## Discussion

WRKY TFs form one of the largest plant-specific TF families, exerting crucial roles in regulating the responses to biotic and abiotic stimuli. In cotton, thus far only several group I and II WRKY TFs have been characterized, while the functional roles of group III WRKY TFs remain elusive. In this study, we isolated a group III WRKY TF gene, *GhWRKY27a*, from cotton (*G. hirsutum*). The presence of conserved motifs and phylogenetic tree analysis further confirmed that *GhWRKY27a* was a member of group III (Figure 1). In addition, the subcellular localization of GhWRKY27a-GFP indicated that the fusion protein was located in the nucleus (Figure 2), which is consistent with previous studies on WRKY TFs from other species (Wang et al., 2013). These results indicate that *GhWRKY27a* may function in the nucleus.

Previous studies have shown that the expression of certain stress-induced proteins is associated with stress tolerance (Huang et al., 2011; Ma et al., 2013; Wang et al., 2014). The expression pattern of a gene is usually an indicator of its function (Li et al., 2015). In this study, the results from qRT-PCR analyses indicated that the transcription of *GhWRKY27a* in cotton was induced not only by abiotic stresses, pathogen infection, but also by multiple signaling molecules such as H_2_O_2_, ABA, SA, MeJA, and ET (Figure 3). These findings suggest that *GhWRKY27a* may function as a regulator that links multiple signaling networks in abiotic and biotic stress adaptation.

To expand our previous analysis of the biological roles of *GhWRKY27a*, we explored the possible contribution of *GhWRKY27a* to abiotic stress responses using drought stress as a model. Our results showed that silencing *GhWRKY27a* in cotton enhanced the tolerance to drought stress (Figure 4) and *GhWRKY27a* overexpression in *N. benthamiana* reduced its tolerance to drought stress (Figure 6), indicating that GhWRKY27a is a negative regulator of tolerance to drought stress. The function of GhWRKY27a in drought stress tolerance is similar to *Arabidoposis* WRKY53, which is phylogenetically related to GhWRKY27a (Dou et al., 2014). It was shown that activated expression of AtWRKY53 negatively regulated drought tolerance (Sun and Yu, 2015). The phytohormone ABA is essential in plant responses to drought stress, and an increased ABA content is beneficial for plants under drought stress as a result of ABA-induced changes at the cellular and whole-plant levels (Xiong and Zhu, 2003). The OE plants accumulated less ABA under drought stress compared with the WT plants (Figure 7C), which indicated that GhWRKY27a functioned in the ABA response in *N. benthamiana*. In addition, the transpirational water loss through the stomata is a key determinant of drought tolerance (Xiong et al., 2002). We found that the ectopic expression of *GhWRKY27a* in OE plants driven by the CaMV35S promoter did not result in the impairment of stomatal closure under normal conditions (Figure 6D). However, the more rapid water loss and impaired stomatal closure were observed in OE plants under drought stress (Figure 6D). We speculated that *GhWRKY27a* may not be expressed in guard cells of stomata, and impair stomatal closure indirectly. The regulation of stomatal movement may result from the interference of GhWRKY27a with the endogenous WRKY regulatory networks. Besides that, compared with normal condition, *GhWRKY27a* may interfere with more endogenous regulators under drought condition. Of course, further research is required to examine whether GhWRKY27a localizes to the guard cells of stomata and affects ABA level. Moreover, the results of this study showed that OE plants antagonistically regulated the expression of ABA- and drought-responsive genes, including *SnRK2.3, AREB, LEA*, and *P5CS* (Figure 7D). Under drought stress conditions, these stress-related genes are induced and are considered to play a role in defense response (Shinozaki and Yamaguchi-Shinozaki, 2007). Based on these data, we infer that *GhWRKY27a* might confer reduced drought tolerance, which was coupled with impaired stomatal closure and downregulated expression of drought-responsive genes. On the other hand, we found that OE plants showed greater accumulation of ROS than WT plants after drought treatment, and *GhWRKY27a* overexpression resulted in reduced tolerance to oxidative stress (Figure 8), indicating that GhWRKY27a is also a negative regulator of tolerance to oxidative stress. It has been hypothesized that ROS production may be the primary symptom of phytotoxicity under abiotic stress (Choudhury et al., 2013). Thus, we infer that GhWRKY27a may function in drought stress by regulating production of ROS. This is similar to the observation that overexpression of *GhWRKY17* in *N. benthamiana* reduced drought stress tolerance by enhancing ROS accumulation (Yan et al., 2014).

In addition to the vital roles of WRKY TFs in abiotic tolerance, their role in disease resistance has been well documented (Cheng et al., 2015; Li et al., 2015). Intriguingly, certain pathogen-responsive group III WRKY genes function to repress plant basal disease resistance. For example, the WRKY domain in the group III WRKY TF AtWRKY52/RRS1 plays a negative role in defense signaling (Le Roux et al., 2015). AtWRKY38 and AtWRKY62 act as transcriptional activators and suppress disease resistance and defense gene expression in *Arabidopsis* (Kim et al., 2008). In this study, our results showed that *GhWRKY27a*-overexpressing plants exhibited enhanced susceptibility to the pathogen *R. solani* as measured by enhanced disease symptoms (Figure 9), implying that GhWRKY27a may play a negative role in the response to pathogen infection. However, there was no obvious difference in morphology between OE and WT plants under normal condition (Figure 6A). It is possible that certain *N. benthamiana* WRKY TFs positively regulate basal disease resistance (Yamamoto et al., 2004; Zhang et al., 2012), and this may interfere with the function of negative regulator of GhWRKY27a under normal condition. After pathogen infection, certain pathogen effectors can selectively target host WRKY TFs to disable defenses, and potentially avoid negative defense components whose inactivation would be disadvantageous for pathogen infection (Le Roux et al., 2015). This is also consistent with the observation that the OE plants were more susceptible to pathogen infection (Figure 9). The interactions between plants and pathogenic microbes are complex, thus further studies regarding WRKY TFs are necessary to parse the interactions between plants and pathogenic microbes.

The accumulation of ROS during infection of plants by necrotrophic pathogens has been implicated in susceptible response against these pathogens (Govrin and Levine, 2000). The results of this study indicated that OE plants demonstrated greater accumulation of ROS than WT plants after inoculation with *R. solani* (Figure 10A), consistent with the up-regulated expression of the ROS-producing genes *RbohA* and *RbohB* and the down-regulated expression of the antioxidant genes encoding SOD, GST, APX, and CAT (Figure 10B). Thus, it is likely that *GhWRKY27a* overexpression altered the expression of oxidation-related genes in response to *R. solani* infection and resulted in the accumulation of ROS, leading to susceptibility to this pathogen. On the other hand, the reduced expression of *HSR515* and *PR* gene (Figures 10B,C), reliable molecular markers of SA-dependent plant defense (Zheng et al., 2006), indicates that *GhWRKY27a* overexpression suppressed SA-mediated defense signaling pathways. Moreover, the JASMONAT-ZIM domain (JAZ) proteins are key repressors of JA signaling and represent a crucial interface in the JA signaling cascade (Pauwels and Goossens, 2011; Kazan and Manners, 2012). Notably, the levels of *NbJAZ1*/*NbJAZ3* were higher in OE plants than in WT plants (Figure 10C), suggesting that GhWRKY27a overexpression attenuated the JA-dependent signaling pathway, which is important for resistance to necrotrophic pathogens (Glazebrook, 2005). This is in agreement with the observations that cotton GbWRKY1 functions as a negative regulator of the JA-mediated defense response and plant resistance to the pathogens *Botrytis cinerea* and *Verticillium dahliae* by activating *JAZ1* expression (Li et al., 2014b). Li et al. (2015) also showed that the enhanced pathogen resistance observed in *SpWRKY1*-overexpressing tobacco plants is correlated with the SA-dependent and JA-dependent defense pathways. Therefore, we hypothesized that the disease susceptibility of OE plants may be related to the repression of SA-dependent and JA-dependent defense pathways.

The increased transcript levels of *GhWRKY27a* under drought stress and pathogen attack is paradoxical, because GhWRKY27a functions in negative responses to drought tolerance and in resistance to *R. solani* infection. Similar results have been reported in other plant species. In soybean, a stress-induced gene, *GmWRKY13*, negatively regulates drought stress responses (Zhou et al., 2008). *C. annuum CaWRKY1*, which is strongly induced by pathogen infections and the signal molecular SA, acts as a negative regulator to prevent spurious activation of defense responses at suboptimal concentrations of SA (Oh et al., 2008). In *Arabidopsis*, Wang et al. (2006) showed that *WRKY58* acts as a negative regulator in defense, and the *wrky58* mutant was more resistant to a pathogen than WT plants after treatment with a suboptimal level of benzothiadizole. We infer that the role of *GhWRKY27a* may be similar to that of these negative regulators, which is to prevent unnecessary activation of defense responses at suboptimal levels of signal molecules. In addition, another possibility is that *GhWRKY27a*, which is induced by multiple stresses, adjusts the intensity of defense responses in cooperation with stress-responsive positive regulators, and prevents overactivation of defense responses whenever stress diminishes. Further studies are needed to elucidate the role of GhWRKY27a in various biological processes.

Based on these data, we conclude that GhWRKY27a exerts important physiological functions in the negative regulation of tolerance to drought stress and resistance against *R. solani* infection, likely through modulating multiple signaling pathways. Although the complex regulatory mechanisms involving group III WRKY proteins in cotton remain unclear, this work provides further insight into the regulatory mechanisms of a group III WRKY protein.

## Author contributions

YY carried out most of the experiments, and drafted the manuscript. HJ participated in qRT-PCR analysis. HJ, FW, CW, and SL helped to revise the manuscript. XG designed the experiments and helped to draft the manuscript. All authors read and approved the final manuscript.

### Conflict of interest statement

The authors declare that the research was conducted in the absence of any commercial or financial relationships that could be construed as a potential conflict of interest.

## Supplementary material

Figure S1 **Schematic representation of the DNA structures**. The lengths of the extrons and introns of *GhWRKY27a* (GenBank accession number: KM453244), *AtWRKY41* (GenBank accession number: NW_003302550.1), *AtWRKY30* (GenBank accession number: NC_003076.8), and *AtWRKY54* (GenBank accession number: NC_003071.7) are shown according to the scale below.

Figure S2 **Silencing efficiency of the *GhWRKY27a* gene silenced plants**. Seven-day-old seedlings were infiltrated with agrobacteria and leaf samples were collected 20 days after VIGS treatment. The silencing efficiencies of *GhWRKY27a* in wild-type (WT), vector control (CK), and *GhWRKY27a* gene silenced (VIGS) plants were analyzed via qRT-PCR. The *ubiquitin* gene (GenBank accession number: EU304080) was employed as an internal control. The data are presented as the mean ± standard error of three independent experiments.

Figure S3 **Characterization of transgenic tobacco plants**. **(A)** The evaluation of transgenic plants in the T_0_ progeny of transgenic plants by PCR. **(B)** Analysis of *GhWRKY27a* expression in wild-type (WT), T_1_ *GhWRKY27a*-overexpressing (OE) plants, and T_3_ *GhWRKY27a*-overexpressing (OE) plants. The *N. benthamiana* β*-actin* gene (GenBank accession number: JQ256516) was used as a loading control.

Table S1 **The primers used for general PCR in this study**.

Table S2 **PCR amplification conditions**.

Table S3 **The primers used for qRT-PCR in this study**.
