# Supplementary figures and images for: Overexpression of GhWRKY27a reduces tolerance to drought stress and resistance to Rhizoctonia solani infection in transgenic Nicotiana benthamiana
Source: Front Physiol. 2015 Sep 24;6:265. doi: 10.3389/fphys.2015.00265 (PMC4586331; doi:10.3389/fphys.2015.00265)

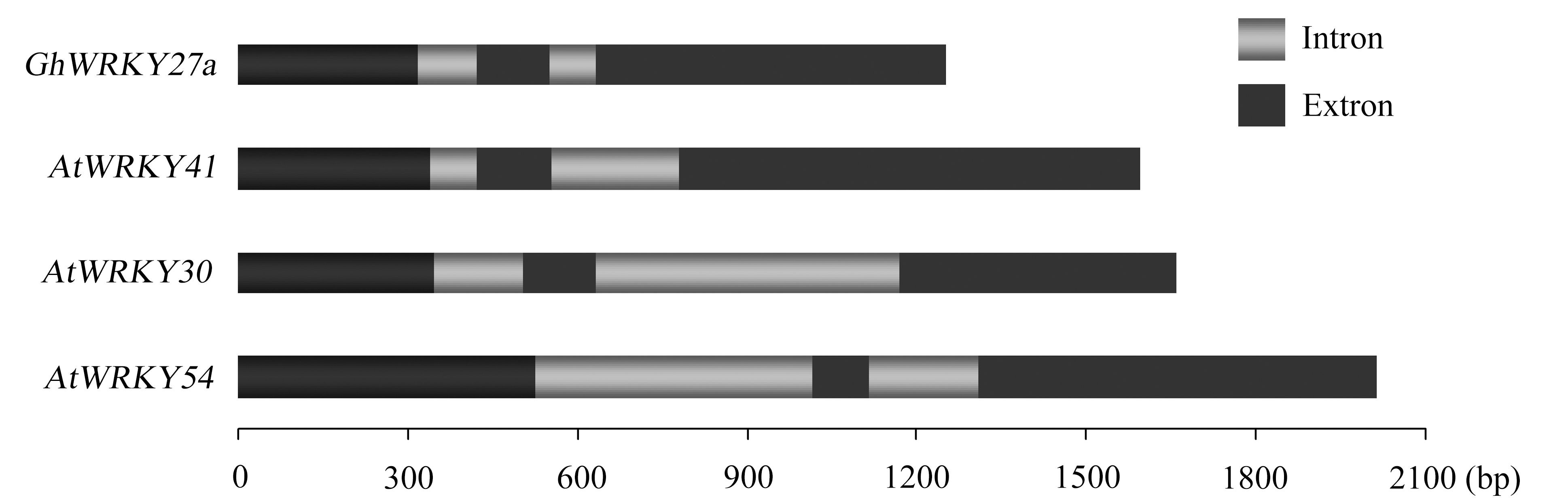

Supplement: Figure S1 — Schematic representation of the DNA structures. The lengths of the extrons and introns of GhWRKY27a (GenBank accession number: KM453244), AtWRKY41 (GenBank accession number: NW_003302550.1), AtWRKY30 (GenBank accession number: NC_003076.8), and AtWRKY54 (GenBank accession number: NC_003071.7) are shown according to the scale below. [file Image1.TIF]

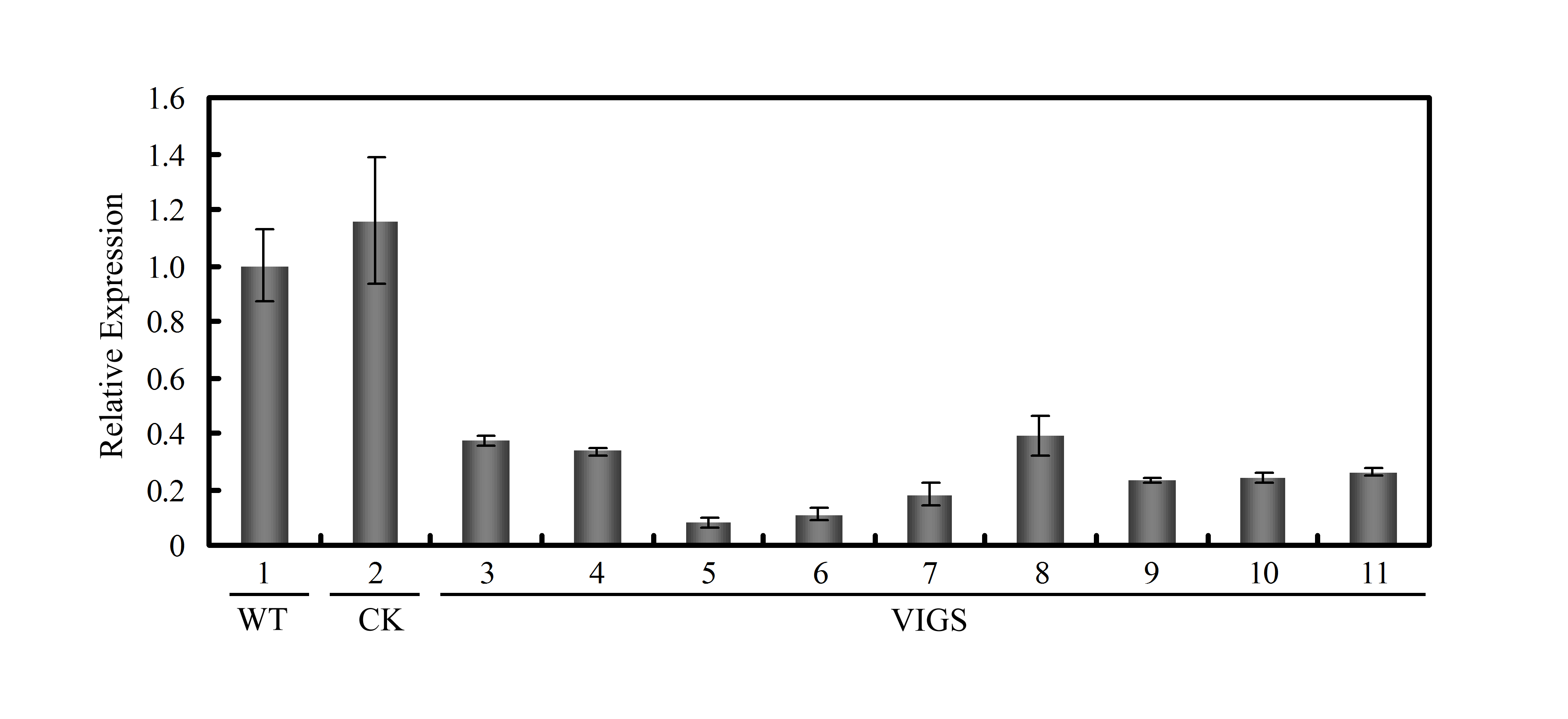

Supplement: Figure S2 — Silencing efficiency of the GhWRKY27a gene silenced plants. Seven-day-old seedlings were infiltrated with agrobacteria and leaf samples were collected 20 days after VIGS treatment. The silencing efficiencies of GhWRKY27a in wild-type (WT), vector control (CK), and GhWRKY27a gene silenced (VIGS) plants were analyzed via qRT-PCR. The ubiquitin gene (GenBank accession number: EU304080) was employed as an internal control. The data are presented as the mean ± standard error of three independent experiments. [file Image2.TIF]

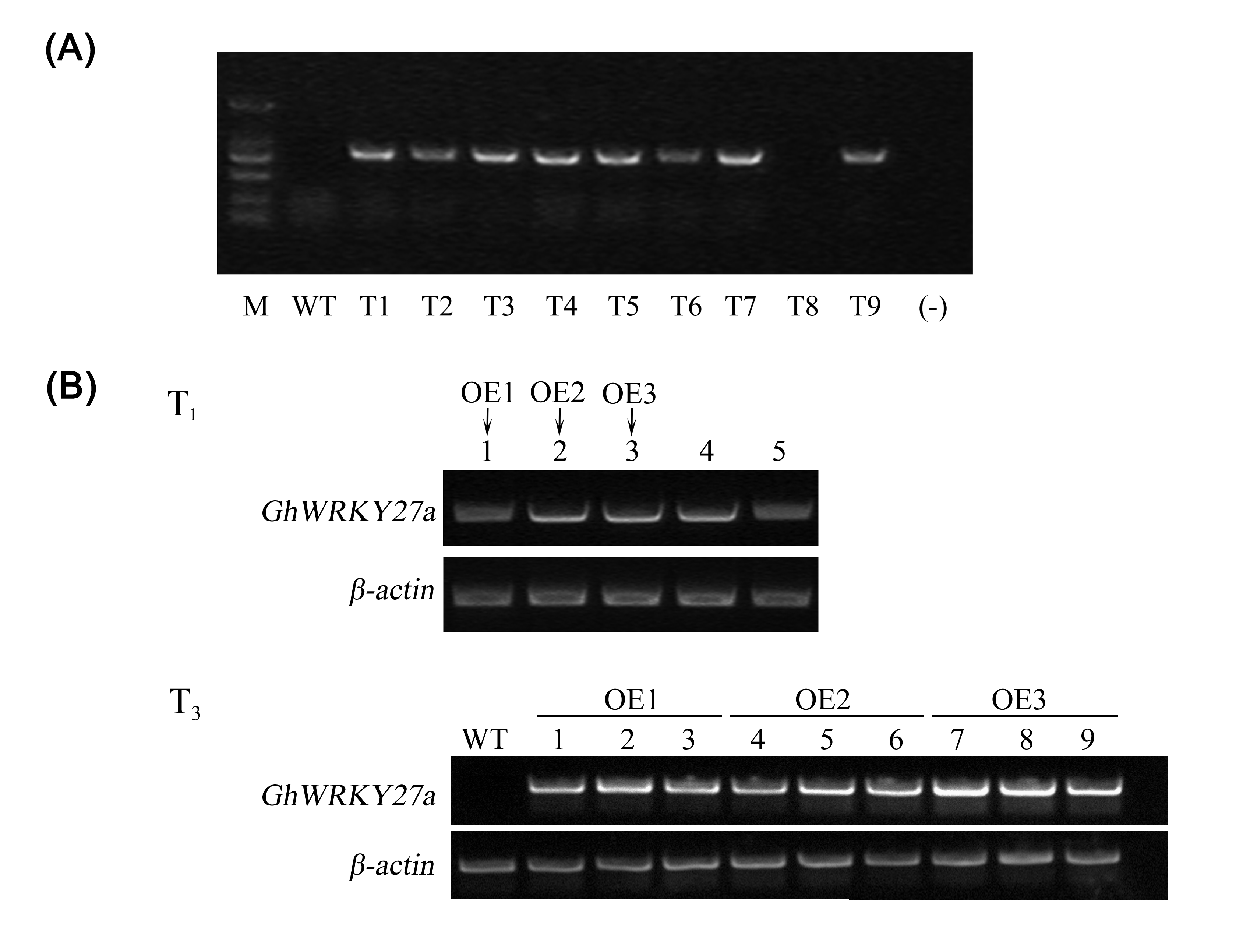

Supplement: Figure S3 — Characterization of transgenic tobacco plants. (A) The evaluation of transgenic plants in the T0 progeny of transgenic plants by PCR. (B) Analysis of GhWRKY27a expression in wild-type (WT), T1 GhWRKY27a-overexpressing (OE) plants, and T3 GhWRKY27a-overexpressing (OE) plants. The N. benthamiana β-actin gene (GenBank accession number: JQ256516) was used as a loading control. [file Image3.TIF]
