# Supplementary material for: Overexpression of GhWRKY27a reduces tolerance to drought stress and resistance to Rhizoctonia solani infection in transgenic Nicotiana benthamiana
Source: Front Physiol. 2015 Sep 24;6:265. doi: 10.3389/fphys.2015.00265 (PMC4586331; doi:10.3389/fphys.2015.00265)
Supplement: Table S1 — The primers used for general PCR in this study. [file Table1.DOC]

Table S1 The primers used for general PCR in this study.

| Abbreviation | Primer sequence (5'-3') | Description |
| --- | --- | --- |
| M1 | GGAGCARARGACAYTGMWWARYGAG | cDNA sequence primer, forward |
| M2 | TCTYTGSACTTGYTTYGTKGCCCA | cDNA sequence primer, reverse |
| 5P1 | CTTCATCTGATCTCTGGACTTG | 5'RACE reverse primer, outer |
| 5P2 | GGGTGTTTCGATAGGTGCATCTG | 5'RACE reverse primer, inner |
| 3P1 | GCTCTGAAAACCTGCTGG | 3'RACE forward primer, outer |
| 3P2 | GGAGAAAATACGGGCAAAAGG | 3'RACE forward primer, inner |
| AAP | GGCCACGCGTCGACTAGTAC(G)_14_ | Abridged Anchor Primer |
| AUAP | GGCCACGCGTCGACTAGTAC | Abridged Universal Amplification Primer |
| B25 | GACTCTAGACGACATCGA | 3'RACE universal primer, outer |
| B26 | GACTCTAGACGACATCGA(T)_18_ | 3'RACE universal primer, inner |
| CS1 | CAGTTGAAGGAAAGAAGAAAGGG | Full-length cDNA sequence primer, forward |
| CS2 | GAACCCGAATTTGACATCTACTG | Full-length cDNA sequence primer, reverse |
| G1 | CCTTTCACTCTCCTACTCCCTC | Genomic sequence primer, forward |
| G2 | GAACCCGAATTTGACATCTACTG | Genomic sequence primer, reverse |
| QS1 | CTTACAGTTGAAGGAAAGAAGAAAGG | IPCR forward primer, outer |
| QS2 | CAACAGAAGATGATGATGCACCC | IPCR reverse primer, outer |
| QS3 | GGGTGCATCATCATCTTCTGTTG | IPCR forward primer, inner |
| QS4 | CTTGAATCAGCTCGCTCACCAGTG | IPCR reverse primer, inner |
| QYZ1 | CAGGTGACCCAACCCTTTGC | Promoter special primer, forward |
| QYZ2 | CTTAACATTCAGGCCAAGTTCCATG | Promoter special primer, reverse |
| ZH1 | TCTAGAATGGAGAACATGTGGAAGTGGGAG | Vector construction primer, forward |
| ZH2 | GTCGACGAACCCGAATTTGACATCTACTG | Vector construction primer, reverse |
| SL1 | TCTAGAATGGAGAACATGTGGAAGTGGGAG | Subcellular localization primer, forward |
| SL2 | CTCGAGGGAGAAAAATCCCGGGGTG | Subcellular localization primer, reverse |
| 35SF | GGGTGCATCATCATCTTCTGTTG | CaMV 35S promoter, forward |
| 35SR | GAAGACGTGGTTTTAACG | CaMV 35S promoter, reverse |
