# Supplementary material for: Overexpression of GhWRKY27a reduces tolerance to drought stress and resistance to Rhizoctonia solani infection in transgenic Nicotiana benthamiana
Source: Front Physiol. 2015 Sep 24;6:265. doi: 10.3389/fphys.2015.00265 (PMC4586331; doi:10.3389/fphys.2015.00265)
Supplement: Table S2 — PCR amplification conditions. [file Table2.DOC]

**Table S2** **PCR amplification conditions.**

| Primer pair | Amplification conditions |
| --- | --- |
| M1/M2 | 10 min at 94 °C, 40 s at 94 °C, 40 s at 48 °C, 60 s at 72 °C for 35 cycles, 10 min at 72 °C |
| 5P1/AAP | 10 min at 94 °C, 40 s at 94 °C, 40 s at 51 °C, 60 s at 72 °C for 35 cycles, 10 min at 72 °C |
| 5P2/AUAP | 10 min at 94 °C, 40 s at 94 °C, 40 s at 54 °C, 60 s at 72 °C for 35 cycles, 10 min at 72 °C |
| 3P1/B26 | 10 min at 94 °C, 40 s at 94 °C, 40 s at 51 °C, 60 s at 72 °C for 35 cycles, 10 min at 72 °C |
| 3P2/B25 | 10 min at 94 °C, 40 s at 94 °C, 40 s at 53 °C, 90 s at 72 °C for 35 cycles, 10 min at 72 °C |
| CS1/CS2 | 10 min at 94 °C, 40 s at 94 °C, 40 s at 50 °C, 90 s at 72 °C for 35 cycles, 10 min at 72 °C |
| G1/G2 | 10 min at 94 °C, 40 s at 94 °C, 40 s at 51 °C, 2 min at 72 °C for 35 cycles, 10 min at 72 °C |
| QS1/QS2 | 10 min at 94 °C, 40 s at 94 °C, 40 s at 47 °C, 2 min at 72 °C for 35 cycles, 10 min at 72 °C |
| QS3/QS4 | 10 min at 94 °C, 40 s at 94 °C, 40 s at 54 °C, 2 min at 72 °C for 35 cycles, 10 min at 72 °C |
| QYZ1/QYZ2 | 10 min at 94 °C, 40 s at 94 °C, 40 s at 53 °C, 2 min at 72 °C for 35 cycles, 10 min at 72 °C |
| ZH1/ZH2 | 10 min at 94 °C, 40 s at 94 °C, 40 s at 57 °C, 40 s at 72 °C for 30 cycles, 10 min at 72 °C |
| SL1/SL2 | 10 min at 94 °C, 40 s at 94 °C, 40 s at 57 °C, 40 s at 72 °C for 30 cycles, 10 min at 72 °C |
| 35SF/35SR | 5 min at 94 °C, 30 s at 94 °C, 30 s at 52 °C, 30 s at 72 °C for 32 cycles, 5 min at 72 °C |
