# Supplementary material for: Overexpression of GhWRKY27a reduces tolerance to drought stress and resistance to Rhizoctonia solani infection in transgenic Nicotiana benthamiana
Source: Front Physiol. 2015 Sep 24;6:265. doi: 10.3389/fphys.2015.00265 (PMC4586331; doi:10.3389/fphys.2015.00265)
Supplement: Table S3 — The primers used for qRT-PCR in this study. [file Table3.DOC]

**Table S3 The primers used for qRT-PCR in this study.**

| Name | Primer sequence (5'-3') |
| --- | --- |
| *GhWRKY27a* | ACACTGGTGAGCGAGCTGATT forward |
|  | TTTTGTTGTGGCTGTTCCGTAG reverse |
| *Ghubiquitin* | CCAGAAGGAATCCACTTTGC forward |
|  | CCAGCTCACATCAGCATACG reverse |
| *Nbβ-actin* | TGGACTCTGGTGATGGTGTC forward |
|  | CCTCCAATCCAAACACTGTA reverse |
| *NbSOD* | GGAGAGCCTTGTCTGATGG forward |
|  | TGGGTCCTGATTAGCAGTGGT reverse |
| *NbGST* | AGCACCCTTACCTTTCCCTC forward |
|  | GCTTTCCTTCACAGCAGCATCA reverse |
| *NbAPX* | CGCTCCTCTTATGCTCCGTCTT forward |
|  | GGTGGCTCTGTCTTGTCCTCTC reverse |
| *NbCAT* | CACAGCCACGCTACTCAAGAC forward |
|  | CCACCCACCGACGAATAAAG reverse |
| *NbRbohA* | ACACACGCCATCAGAACTCCA forward |
|  | CCCACCCAACCAAAATACGC reverse |
| *NbRbohB* | GTTTGCCAGCCACCACCTAAT forward |
|  | AAGAGCAGAACGAGCATCACC reverse |
| *NbAREB* | TTGCTGGTGGAAATGTAAGTGC forward |
|  | GGAATGTAACATCCTTTGAGTATCG reverse |
| *NbHSR515* | TTGGGCAGAATAGATGGGTA forward |
|  | TTTGGTGAAAGTCTTGGCTC reverse |
| *NbPR1a* | GGTGTAGAACCTTTGACCTGG forward |
|  | GAACCCTAGCACATCCAACAC reverse |
| *NbPR1c* | CTTGTCTCTACGCTTCTC forward |
|  | AACACGAACCGAGTTACG reverse |
| *NbPR2* | ACCATCAGACCAAGATGT forward |
|  | TGGCTAAGAGTGGAAGGT reverse |
| *NbACS6* | GCATTGTTATGAGTGGAGGGG forward |
|  | CAGATTCTAAGGCTTCTTTTGTGAC reverse |
| *NbJAZ1* | GTCACCGGCCAGAAGTCTC forward |
|  | TGGCACCTGAGTTCGCGTAC reverse |
| *NbJAZ3* | CTGAGGCAAAATCTGAACCGGAG forward |
|  | GCACCCAATCCAAGCCACAC reverse |
| *NbNPR1* | GCAGCAGACGATGTAATGATGG forward |
|  | TCCACAAGCCTAGTGAGCCTC reverse |
| *NbSnRK2.3* | GGCTAGGCTAGTTAAGGACAAGA forward |
|  | GTTCTCCACCTGCTGCATACTCC reverse |
| *NbLEA* | GGATCTAATTGACAAGGCGAAG forward |
|  | CTCGCCGCTATAAGAGAGAG reverse |
| *NbP5CS* | GTTACCATACCACGTCCCATAG forward |
|  | GAGCTAGGACGCTCCATATTT reverse |
